# Association of high-density lipoprotein-related inflammatory indicators with diabetic foot ulcer in patients with diabetes: a population-based study

**DOI:** 10.1186/s13098-025-01962-8

**Published:** 2025-09-23

**Authors:** Renhe Deng, Ruyi Tao, Huiyi Luo, Yueqi Gu, Mengyu Yang, Chaoqi Yin

**Affiliations:** 1 Department of Plastic Surgery and Burn, Third Xiangya Hospital, Central South University, Changsha, 410013 Hunan China; 2 Department of Burns and Plastic Surgery, The People’s Hospital of Xiangxi Autonomous Prefecture, Jishou, 416000 Hunan China

**Keywords:** Diabetic foot, Lipoproteins, HDL, Neutrophils, Monocytes, Lymphocytes, Blood platelets

## Abstract

**Background:**

Early identification and treatment of diabetic foot ulcer (DFU) in diabetes mellitus (DM) patients is of great importance for improving life quality. This study aimed to investigate the association between high-density lipoprotein (HDL)-related inflammatory indicators, such as neutrophils-to-HDL ratio (NHR), monocyte-to-HDL ratio (MHR), lymphocyte-to-HDL ratio (LHR), platelet-to-HDL ratio (PHR), and the occurrence of DFU in DM patients.

**Methods:**

This study included 1211 DM patients from the National Health and Nutrition Examination Survey (1999–2004). The relationship between HDL-related inflammatory indicators and DFU was explored with logistic regression models. Using a threshold effects analysis model, the association and inflection points between HDL-related inflammatory indicators and diabetic foot ulcer were investigated. Subgroup analyses were performed to further confirm the relationship in different populations. Mediation analysis was conducted to examine how red blood cell mediates the relationship between HDL-related inflammatory indicators and DFU.

**Results:**

After multivariable adjustment, there is a strongly positive relationship between NHR, MHR, PHR, and DFU, whereas no such associations were found between LHR and DFU. Threshold effect analysis showed an inflection point of 0.29 between MHR and DFU, with a 4.51-fold increase in the prevalence of DFU for each unit rise in MHR when MHR was more than the inflection point. Mediation analysis revealed that red blood cell partially mediates the association between NHR and DFU.

**Conclusions:**

These findings reveal a clear association between NHR, MHR, PHR, and an increased prevalence of DFU, which can be used as potential biomarkers in the prevention and management of DFU.

**Supplementary Information:**

The online version contains supplementary material available at 10.1186/s13098-025-01962-8.

## Introduction

Diabetes mellitus (DM), with an increase of 630 million patients from 1990, is currently a public health challenge, leading to economic and social burden across countries [1]. It is expected that the prevalence of diabetes will continue to rise due to factors such as obesity, smoking, ageing, and so on [2–4]. As one of the most severe peripheral neuropathy and arterial complications of DM, the underlying mechanism of delayed healing in diabetic foot ulcer (DFU) is often associated with persistent infection, excessive inflammation, and impaired angiogenesis [5, 6]. The overall mortality of DFU can reach nearly 50% mortality within 5 years, while it is also a leading cause of non-traumatic lower-extremity amputations, representing a major clinical challenge to the survival of patients [7–9]. Consequently, early identification and treatment of DFU is of great importance for improving the quality of life for patients.

High-density lipoprotein (HDL), which is often regarded as “good cholesterol” due to its various protective functions, including anti-inflammatory and antioxidant effects, can be associated with the occurrence and progression of a lot of diseases, such as metabolic syndrome and cardiovascular diseases (CVDs) [10]. Specifically, a large-scale population-based study suggests that HDL acts as an independent protective factor against peripheral artery disease (PAD) in patients with DM. This protective effect may, in turn, contribute to a lower risk of developing DFU [11]. Moreover, a machine learning-based study indicates that HDL can be a key predictor for DFU in patients with diabetic peripheral neuropathy [12].

Complete blood count (CBC), consisting of leukocytes, lymphocytes, neutrophils, monocytes, and other blood components, is a common marker for infection, inflammation, and immune function, which can be used to predict the prognosis of diseases [13, 14]. Previous researches demonstrate the relationship between different CBC-derived inflammatory markers and DFU. In a large population study, monocyte-to-lymphocyte ratio (MLR), neutrophil-to-lymphocyte ratio (NLR), neutrophil-monocyte-to-lymphocyte ratio (NMLR), and systemic inflammatory response index (SIRI) showed significant positive correlations with diabetic foot risk [15]. In a matched case-control study, the investigators found that NLR, platelet‒lymphocyte ratio (PLR), and lymphocyte‒white blood cell ratio (LWR) may have value as a predictive marker for amputation in patients with DFU [16]. However, no existing studies were conducted to determine the association between the HDL-related inflammatory indicators, which combined with HDL and CBC, such as neutrophils-to-HDL ratio (NHR), monocyte-to-HDL ratio (MHR), lymphocyte-to-HDL ratio (LHR), platelet-to-HDL ratio (PHR), and occurrence of DFU in individuals with diabetes.

In this study, we aimed to explore the association between NHR, MHR, LHR, PHR, and DFU from data in the National Health and Nutrition Examination Survey (NHANES) for the first time.

## Materials and methods

### Study design and population

NHANES is a nationwide survey conducted by the National Center for Health Statistics (NCHS), a part of the Centers for Disease Control and Prevention (CDC). The survey involves home health interviews and physical examinations in fully equipped mobile medical centers. Additionally, urine and blood samples are collected from the participants to analyze their health status further. The research protocol was reviewed and approved by the NCHS Ethics Review Board at the CDC, and written informed consent was obtained from all participants.

**Fig. 1:**
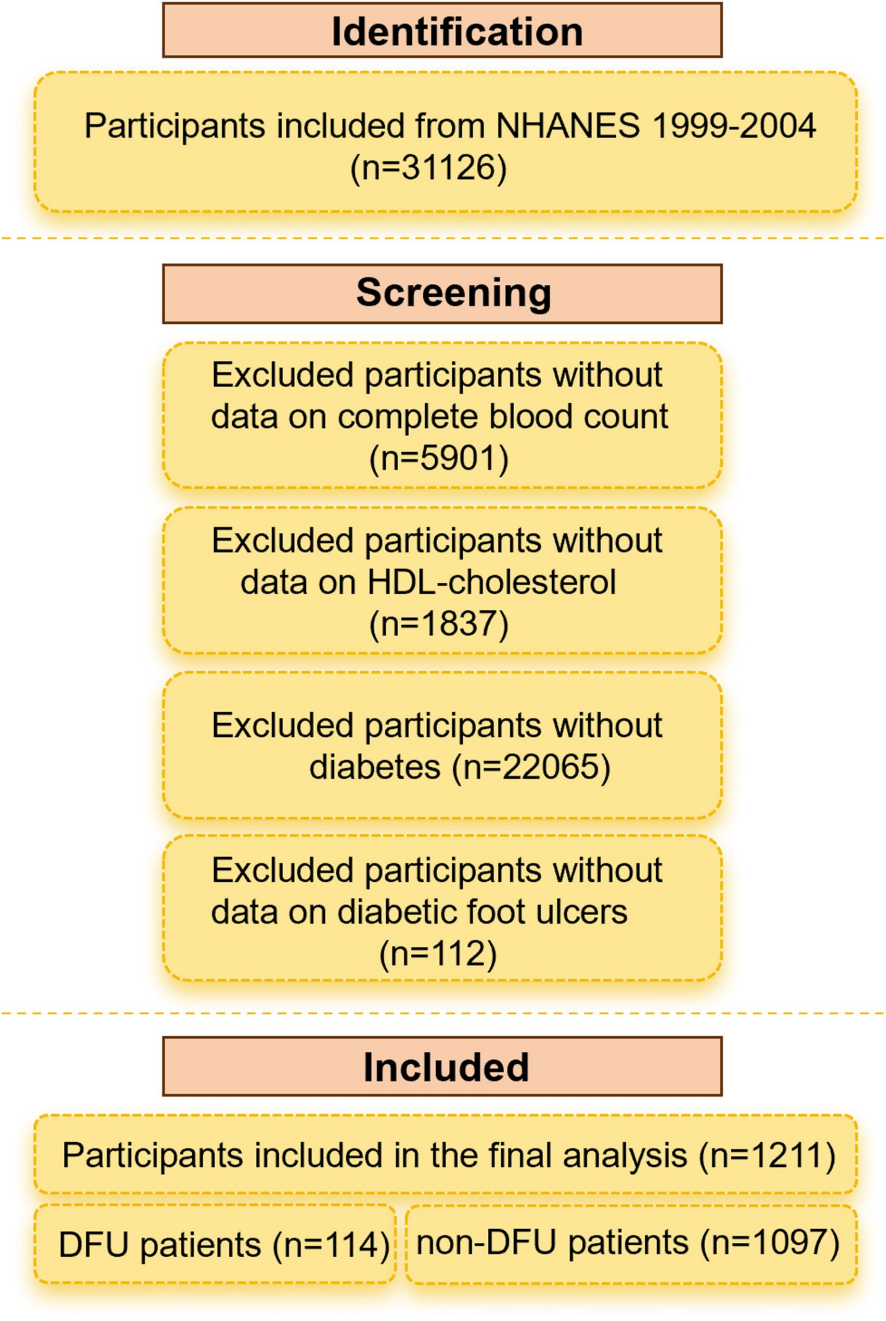
Flowchart of the selection process for study participants from the National Health and Nutrition Examination Survey (NHANES)

In the study, we excluded participants with missing data on complete blood count (*n* = 5901) or who did not have data on HDL-cholesterol (*n* = 1,837), without a diagnosis of DM (*n* = 22065), or who did not have data on diabetic foot ulcers (*n* = 112). The final analytic sample included 1211 diabetes patients aged 40–85 years, including 114 DFU patients and 1097 non-DFU patients, as shown in Fig. 1.

### Measurement of HDL-related inflammatory indicators

Blood specimens were measured at the NHANES Mobile Examination (MEC) Centers. The methods used to derive CBC parameters are based on the Beckman Coulter^®^ method of counting and sizing, in combination with an automatic diluting and mixing device for sample processing, and a single beam photometer for hemoglobinometry. The white blood cells (WBC) differential uses VCS technology. Meanwhile, the blood specimens used for HDL measurement were shipped to the Johns Hopkins University Lipoprotein Analytical Laboratory for analysis. The following HDL-related inflammatory indicators were calculated: NHR, MHR, LHR, and PHR using the following formulas: NHR = neutrophil counts/high-density lipoprotein concentration, MHR = monocyte counts/high-density lipoprotein concentration, LHR = lymphocyte counts/high-density lipoprotein concentration, and PHR = platelet counts/high-density lipoprotein concentration.

### Definition of diabetes and diabetic foot ulcer

Diabetes was characterized as follows: (1) Having received a medical diagnosis of diabetes by a physician. (2) Fasting blood glucose ≥ 7.0 mmol/L. (3) hemoglobin A1c (HbA1c) ≥ 6.5%. (4) Random blood glucose ≥ 11.1 mmol/L. (5) Administration of antidiabetic drugs. Fulfilling any of these criteria was considered as having diabetes. DFU was identified through survey findings. Specifically, DFU presence was identified through participants’ responses to the question, “Do you have any sores or ulcers on your legs or feet that take more than four weeks to heal?” Participants who answered “Yes” were categorized as having DFU, while those who answered “No” were categorized as not having DFU.

### Covariates

Potential confounding factors influencing DFU were carefully considered based on the reference. The covariates considered included age, BMI, gender (male or female), race (White or Non-white), marital status (married or living with a partner, widowed, divorced or separated, never married), family PIR (poverty income ratio), health insurance (Yes or No), CVD (Yes or No), smoking status (every day, some days, not at all), alcohol use, HbA1c, insulin, FPG, CRP, albumin, creatinine and HDL. Supplementary Table S1 offered a specific categorization of the variables.

### Statistical analysis

Participants were separated into two groups based on whether they had DFU. Student t-tests and Chi-Square tests tested baseline variable differences. The relationship between HDL-related inflammatory indicators and diabetic foot ulcer was explored with logistic regression models. Model 1, no covariate was adjusted; Model 2 was adjusted for age, gender, and race. Model 3 added to model 2 the marital status and PIR as covariates. Using a threshold effects analysis model, the association and inflection points between HDL-related inflammatory indicators and diabetic foot ulcer were investigated. Subgroup analyses were conducted in terms of age (< 65, ≥ 65), gender (male, female), race(White, Non-white), PIR (< 1.22, 1.22–2.62, and 2.64-5.00), marital status (married or living with a partner, widowed, divorced or separated, never married). Mediation analysis was performed using ‘mediation” in R with 1,000 bootstrap iterations to examine how red blood cell mediates the relationship between HDL-related inflammatory indicators and diabetic foot ulcer. The statistical analyses were conducted using R (Version 4.4.2) and EmpowerStats (version 2.0). A p-value < 0.05 was determined to be significant.

## Results

### Baseline characteristics of participants

The baseline characteristics of the participants are presented in Table 1. A total of 1,211 adults aged 40–85 years were included in this study, with 114 diagnosed with DFU. The DFU and non-DFU groups displayed some different baseline characteristics, participants with DFU were more likely to have higher BMI and have more CVD events. Among DFU patients, there were significantly higher levels of urine albumin, WBC, neutrophils, basophils, NHR, MHR, PHR, and lower levels of red blood cells (RBCs) and hemoglobin.

**Table 1:** Baseline characteristics of the study population

Characteristics	Total (*n* = 1211)	non-DFU (*n* = 1097)	DFU (*n* = 114)	*P* value
Age (years)	64.75 ± 11.56	64.76 ± 11.55	64.61 ± 11.73	0.897
BMI (kg/m^2^)	30.89 ± 6.56	30.79 ± 6.48	32.03 ± 7.22	0.072
Gender (%)				0.391
Male	623 (51.45%)	560 (51.05%)	63 (55.26%)	
Female	588 (48.55%)	537 (48.95%)	51 (44.74%)	
Race/Ethnicity (%)				0.460
White	471 (38.89%)	423 (38.56%)	48 (42.11%)	
Non-White	740 (61.11%)	674 (61.44%)	66 (57.89%)	
Marital status (%)				0.188
Married/Living with a partner	712 (60.70%)	653 (61.55%)	59 (52.68%)	
Widowed/Divorced/Separated	393 (33.50%)	348 (32.80%)	45 (40.18%)	
Never married	68 (5.80%)	60 (5.66%)	8 (7.14%)	
Family PIR	2.22 ± 1.47	2.24 ± 1.48	2.06 ± 1.40	0.225
Health insurance				0.771
Yes	1073 (89.49%)	968 (89.05%)	105 (92.11%)	
No	126 (10.51%)	117 (10.76%)	9 (7.89%)	
CVD				0.017
Yes	371 (30.76%)	323 (29.55%)	48 (42.48%)	
No	835 (69.24%)	770 (70.45%)	65 (57.52%)	
Smoking status				0.701
Every day	167 (26.05%)	149 (25.60%)	18 (30.51%)	
Some days	31 (4.84%)	28 (4.81%)	3 (5.08%)	
Not at all	443 (69.11%)	405 (69.59%)	38 (64.41%)	
Alcohol use (days)	3.12 ± 21.92	3.29 ± 23.00	1.51 ± 3.64	0.476
HbA1c (%)	7.47 ± 1.83	7.46 ± 1.80	7.59 ± 2.11	0.479
Insulin, (uU/mL)	24.80 ± 33.35	24.95 ± 34.22	23.16 ± 21.76	0.725
FPG, (mg/dL)	166.07 ± 71.77	164.67 ± 69.84	181.46 ± 89.75	0.125
CRP, (mg/dL)	0.71 ± 1.55	0.70 ± 1.57	0.85 ± 1.26	0.300
Albumin, urine (mg/L)	260.12 ± 1124.99	240.04 ± 1100.98	466.15 ± 1335.65	0.050
Creatinine, urine (mg/dL)	110.95 ± 72.96	111.21 ± 72.70	108.35 ± 75.79	0.703
HDL, mg/dL	1.24 ± 0.36	1.24 ± 0.36	1.22 ± 0.41	0.538
CBC count, 10^3^/µL				
White blood cell	7.47 ± 2.18	7.43 ± 2.12	7.87 ± 2.67	0.040
Lymphocyte	2.14 ± 0.88	2.15 ± 0.87	2.05 ± 0.95	0.268
Monocyte	0.59 ± 0.20	0.58 ± 0.20	0.61 ± 0.23	0.095
Neutrophils	4.49 ± 1.66	4.44 ± 1.61	4.92 ± 2.05	0.004
Eosinophils	0.21 ± 0.15	0.21 ± 0.15	0.24 ± 0.14	0.074
Basophils	0.04 ± 0.05	0.04 ± 0.05	0.05 ± 0.06	0.047
Red blood cell	4.60 ± 0.53	4.62 ± 0.52	4.44 ± 0.52	< 0.001
Hemoglobin (g/dL)	13.95 ± 1.59	13.99 ± 1.58	13.61 ± 1.64	0.014
Platelet	254.80 ± 74.35	253.84 ± 73.70	263.95 ± 80.07	0.167
HDL-related inflammatory indicators				
NHR	3.93 ± 1.87	3.87 ± 1.79	4.51 ± 2.46	< 0.001
MHR	0.51 ± 0.23	0.51 ± 0.22	0.57 ± 0.29	0.009
LHR	1.86 ± 0.95	1.86 ± 0.95	1.83 ± 0.92	0.768
PHR	219.84 ± 85.76	218.14 ± 84.55	236.11 ± 95.59	0.033

BMI, Body Mass Index; PIR, poverty income ratio; CVD, cardiovascular disease; HbA1c, hemoglobin A1c (Glycohemoglobin); FPG, fasting plasma glucose; CRP, C-reactive protein; HDL, high-density lipoprotein; CBC, complete blood cell; NHR, neutrophils-to-HDL ratio; MHR, monocyte-to-HDL ratio; LHR, lymphocyte-to-HDL ratio; PHR, platelet-to-HDL ratio

### Association of HDL-related inflammatory indicators and diabetic foot ulcer

**Fig. 2:**
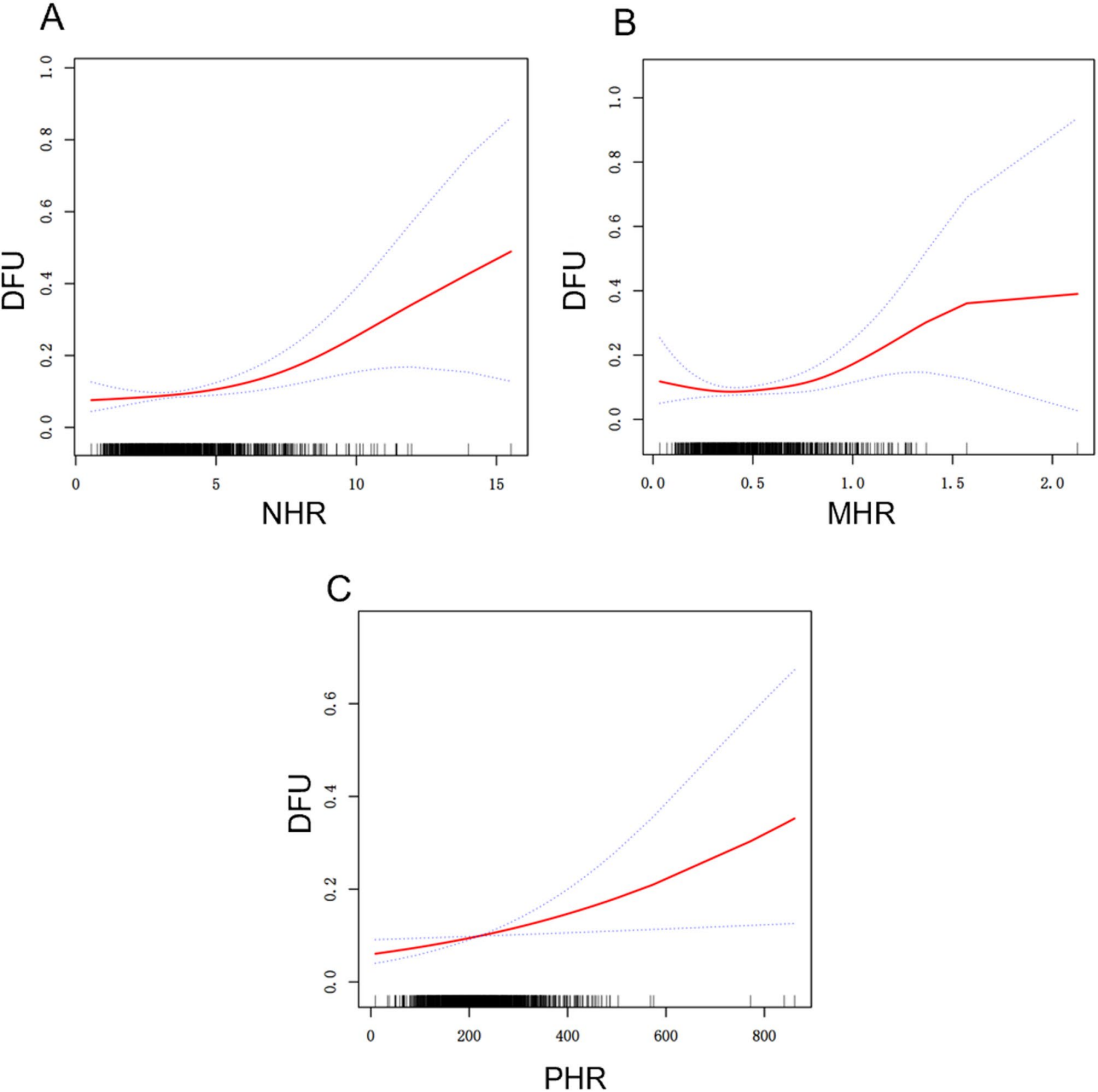
Adjusted smooth spline of non-linear relationship between high-density lipoprotein-related inflammatory indicators and diabetic foot ulcers. (**A**) NHR and DFU. (**B**) MHR and DFU. (**C**) PHR and DFU. The red solid line indicates the smooth curve fit, while the blue band depicts the 95% confidence interval. The model accounted for gender, age, race, marital status, and PIR. NHR, neutrophils-to-HDL ratio; MHR, monocyte-to-HDL ratio; PHR, platelet-to-HDL ratio; DFU, diabetic foot ulcer

Three models were constructed to examine the relationship between HDL-related inflammatory indicators and diabetic foot ulcer in this study (Table 2). In model 1, positive associations between NHR (OR = 1.17, 95% CI = 1.07–1.28, *p* = 0.0005), MHR (OR = 2.71, 95% CI = 1.28–5.77, *p* = 0.0095), PHR (OR = 1.00, 95% CI = 1.00–1.00, *p* = 0.0341) and DFU were observed. After adjustment for gender, age and race/ethnicity in model 2, the positive associations between NHR (OR = 1.16, 95% CI = 1.06–1.27, *p* = 0.0019), MHR (OR = 2.44, 95% CI = 1.11–5.35, *p* = 0.0259), PHR (OR = 1.00, 95% CI = 1.00–1.00, *p* = 0.0460) and DFU were remained. Furthermore, in model 3 adjusted for gender, age, race/ethnicity, marital status and poverty income ratio, the positive associations between NHR (OR = 1.16, 95% CI = 1.06–1.28, *p* = 0.0022), MHR (OR = 2.68, 95% CI = 1.20–5.98, *p* = 0.0161), PHR (OR = 1.00, 95% CI = 1.00–1.00, *p* = 0.0241) and DFU were still significant. When HDL-related inflammatory indicators was divided into 3 quartiles, with the Q1 group as the reference, the risk of DFU increased with NHR Q3 group (OR = 1.66, 95% CI = 1.03–2.66, *p* = 0.0364) in model 1 and the risk of DFU increased with PHR Q3 group in three models (Model 1: OR = 1.68, 95% CI = 1.05–2.67, *p* = 0.0290; Model 2: OR = 1.64, 95% CI = 1.02–2.64, *p* = 0.0423; Model 3: OR = 1.80, 95% CI = 1.08-2 = 3.00, *p* = 0.0233). As illustrated in Fig. 2, potential nonlinear association between HDL-related inflammatory indicators and DFU were investigated, NHR (Fig. 2.A), MHR (Fig. 2.B) and PHR (Fig. 2.C) level showed a statistically significant non-linear association with DFU, which indicated that the prevalence of DFU was increased for every unit rise in the above three indicators. Threshold effect analysis showed an inflection point of 0.29 between MHR and DFU, with a 4.51-fold increase in the prevalence of DFU for each unit rise in MHR when MHR was more than the inflection point (Table 3).

**Table 2:** The association between high-density lipoprotein-related inflammatory indicators with diabetic foot ulcers

Exposure	Model 1	Model 2	Model 3
	OR (95% CI) *p*-Value	OR (95% CI) *p*-Value	OR (95% CI) *p*-Value
NHR	1.17 (1.07, 1.28) 0.0005	1.16 (1.06, 1.27) 0.0019	1.16 (1.06, 1.28) 0.0022
NHR tertile			
Q1(0.52–2.97)	1.0	1.0	1.0
Q2(2.98–4.37)	1.10 (0.66, 1.83) 0.7058	1.02 (0.61, 1.72) 0.9310	1.06 (0.61, 1.84) 0.8291
Q3(4.38–15.52)	1.66 (1.03, 2.66) 0.0364	1.51 (0.93, 2.47) 0.0975	1.56 (0.93, 2.61) 0.0928
MHR	2.71 (1.28, 5.77) 0.0095	2.44 (1.11, 5.35) 0.0259	2.68 (1.20, 5.98) 0.0161
MHR tertile			
Q1(0.03–0.39)	1.0	1.0	1.0
Q2(0.40–0.57)	1.08 (0.66, 1.77) 0.7670	1.02 (0.62, 1.69) 0.9409	1.12 (0.65, 1.91) 0.6828
Q3(0.58–2.13)	1.43 (0.90, 2.29) 0.1339	1.32 (0.82, 2.15) 0.2541	1.48 (0.89, 2.47) 0.1292
LHR	0.97 (0.79, 1.20) 0.7681	0.95 (0.77, 1.18) 0.6635	1.00 (0.79, 1.26) 0.9724
LHR tertile			
Q1(0.31–1.38)	1.0	1.0	1.0
Q2(1.39–2.08)	0.89 (0.55, 1.43) 0.6299	0.87 (0.54, 1.41) 0.5766	0.99 (0.60, 1.65) 0.9796
Q3(2.09–14.62)	0.94 (0.59, 1.50) 0.7939	0.90 (0.56, 1.46) 0.6816	1.02 (0.61, 1.69) 0.9474
PHR	1.00 (1.00, 1.00) 0.0341	1.00 (1.00, 1.00) 0.0460	1.00 (1.00, 1.00) 0.0241
PHR tertile			
Q1(9.30-178.57)	1.0	1.0	1.0
Q2(178.95-241.18)	0.97 (0.58, 1.62) 0.9037	0.95 (0.57, 1.60) 0.8566	1.12 (0.65, 1.94) 0.6847
Q3(241.23–861.40)	1.68 (1.05, 2.67) 0.0290	1.64 (1.02, 2.64) 0.0423	1.80 (1.08, 3.00) 0.0233

Model 1: unadjusted; Model 2: adjusted for gender, age and race/ethnicity; Model 3: adjusted for gender, age, race/ethnicity, marital status and poverty income ratio. NHR, neutrophils-to-HDL ratio; MHR, monocyte-to-HDL ratio; LHR, lymphocyte-to-HDL ratio; PHR, platelet-to-HDL ratio; OR, odds ratio; 95% CI, 95% confidence interval

**Table 3:** Threshold effect analysis of MHR on diabetic foot ulcers

	Adjusted OR (95% CI), *p* Value
MHR	
Inflection point	0.29
<0.29	0.00 (0.00, 0.22) 0.0134
≥ 0.29	4.51 (1.95, 10.43) 0.0004
*P* for likelihood test	0.009

Age, race, BMI, marital status and PIR were adjusted. MHR, monocyte-to-HDL ratio; 95% CI, 95% confidence interval; OR, odds ratio

### Subgroup analysis

To further investigate the impact of the positive association between NHR, MHR, PHR, and DFU in different populations, subgroup analysis and interaction tests were performed based on age, gender, PIR, race, and marital status. For NHR (Supplementary Table S2), this relationship was significant among participants aged 40–64 (OR = 1.27, 95% CI = 1.11–1.46, *p* = 0.0007), male (OR = 1.16, 95% CI = 1.02–1.31, *p* = 0.0187), PIR (1.22–2.62: OR = 1.28, 95% CI = 1.07–1.52, *p* = 0.0065; 2.64-5.00: OR = 1.38, 95% CI = 1.14–1.67, *p* = 0.0011), race (White: OR = 1.23, 95% CI = 1.06–1.42, *p* = 0.0069; Non-White: OR = 1.13, 95% CI = 1.00-1.28, *p* = 0.0439, *p* = 0.0439), Married/Living with a partner (OR = 1.27, 95% CI = 1.12–1.44, *p* = 0.0002). Significant interaction effects were found in PIR and marital status groups. For MHR (Supplementary Table S3), this relationship was significant among participants aged 65–85 (OR = 4.00, 95% CI = 1.25–12.79, *p* = 0.0192), male (OR = 5.16, 95% CI = 1.77–15.01, *p* = 0.0026), White (OR = 6.28, 95% CI = 1.78–22.18, *p* = 0.0043), married/Living with a partner (OR = 5.38, 95% CI = 1.83–15.82, *p* = 0.0022). Significant interaction effects were found in gender group. For PHR (Supplementary Table S4), this relationship was significant among participants aged 40–64 (OR = 1.00, 95% CI = 1.00-1.01, *p* = 0.0158), PIR (1.22–2.62: OR = 1.00, 95% CI = 1.00-1.01, *p* = 0.0412; 2.64-5.00: OR = 1.00, 95% CI = 1.00-1.01, *p* = 0.0185), Married/Living with a partner (OR = 1.00, 95% CI = 1.00-1.01, *p* = 0.0067). No significant interaction effects were found among subgroups.

### Mediation analysis

**Fig. 3:**
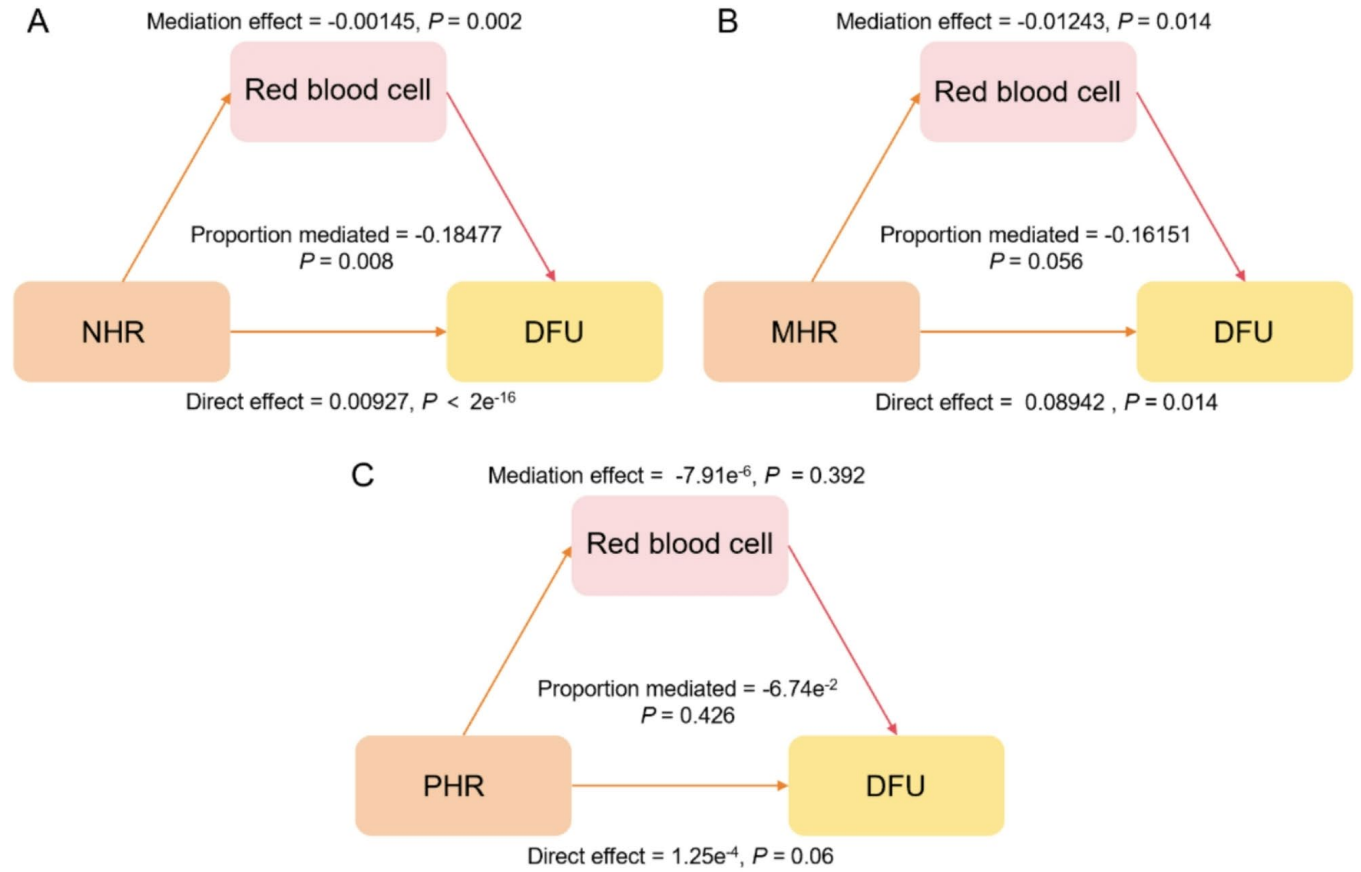
The mediation role of red blood cells in the association between high-density lipoprotein-related inflammatory indicators and DFU. (**A**) NHR (**B**) MHR (**C**) PHR. Adjusted for gender, age, race/ethnicity. NHR, neutrophils-to-HDL ratio; MHR, monocyte-to-HDL ratio; PHR, platelet-to-HDL ratio; DFU, diabetic foot ulcer

RBCs were the hypothesized mediating variable in our non-parametric bootstrapping (1,000 simulations) mediation analysis, which aimed to elucidate the mechanisms behind the relationship between NHR, MHR, PHR, and DFU. Our findings show that the relationship between NHR and DFU is partially mediated by RBCs (Fig. 3). Both a significant direct effect (average direct effect = 0.00927, *p* = 2e^−16^) and a significant indirect effect mediated by red blood cells (average causal mediation effect = − 0.00145, *p* = 0.002) were included in the significant overall positive association between NHR and DFU (total effect = 0.00783, *p* = 0.006). Partial mediation was shown by the 18.47% (*p* = 0.008) of the total impact that was mediated by RBCs.

## Discussion

In the present population-based study, the associations between HDL-related inflammatory indicators and DFU prevalence were explored using data from the NHANES 1999–2004 cycles. To the best of our knowledge, this is the first attempt to assess the relationship between this particular indicator and DFU. The results indicate that there is a positive relationship between NHR, MHR, PHR, and DFU. No such associations were found between LHR and DFU. These findings provide new insights into the prevention and management of complications in people with DM.

HDL-related inflammatory indicators, as novel biomarkers that integrate both lipid metabolism and inflammation status, were discovered the potential clinical value in the context of various diseases and different population groups [17, 18]. In a population-based observational study of 1040 stroke patients and 26,261 healthy controls, per standard deviation (SD) increase in the PHR was associated with an OR of 1.13 (95% CI: 1.03–1.24, *P* = 0.01) for stroke after full adjustment for established risk factors. Further analysis revealed that when the PHR exceeded 223.684, a significantly stronger association with stroke was observed in the noncancer population. The PHR also remained a significant predictor of CVD mortality after adjusting for demographic characteristics, lifestyle factors, and comorbidities [19]. A retrospective study demonstrated that both LHR and NHR were independently associated with the risk of in-stent restenosis in patients undergoing elective percutaneous coronary intervention (PCI), indicating their potential prognostic utility in PCI outcomes [20]. Similarly, elevated MHR was identified as an independent and clinically useful predictor of in-stent restenosis among patients with non-ST segment elevation acute coronary syndrome following drug-eluting stent implantation [21]. Other studies have highlighted the predictive value of HDL-related inflammatory markers in various conditions, including periodontitis, bipolar disorder, and mental health [22–24]. Our findings further support the relevance of these indices by confirming a nonlinear relationship between HDL-related markers and chronic foot ulceration in patients with DM, underscoring the broad applicability and interpretability of HDL-based inflammatory indicators across clinical contexts. The initiation and progression of DFU involve multifaceted mechanisms, including endocrine dysfunction, chronic inflammation, immune dysregulation, and impaired angiogenesis, all of which collectively impair the wound healing process. Current evidence highlights the roles of various cytokines, lipid metabolites, and immune cells in regulating wound repair and regeneration [25–28]. In this context, our findings, which indicate a positive relationship between NHR, MHR, PHR, and DFU, suggest that HDL-related inflammatory indices may reflect key aspects of these pathological processes. For instance, neutrophils contribute to infection control through phagocytosis but may also delay healing via excessive release of reactive oxygen species and pro-inflammatory cytokines when dysregulated [29]. A higher NHR may thus indicate heightened neutrophil activity coupled with reduced anti-inflammatory capacity of HDL. Similarly, alterations in monocyte subsets and lipid profiles, such as the reduction in non-classical monocytes and vitamin D3 levels observed in DFU patients with peripheral artery disease [30], may be captured by MHR, reinforcing its relevance in immune-metabolic dysregulation. Moreover, platelet activation, implicated in DFU through mechanisms such as modulation of microRNAs (e.g., miR-155) via platelet-rich fibrin [31], may be reflected in PHR, linking platelet-derived inflammation and HDL dysfunction. Although LDL has been identified as a critical biomarker for DFU risk [32], our results emphasize the additional prognostic value of HDL-based ratios. Nonetheless, the precise mechanisms through which NHR, MHR, and PHR influence DFU development warrant further investigation to better understand their roles within this complex pathological network.

The results of our subgroup analysis revealed several noteworthy findings that warrant further discussion. Notably, the association between NHR and DFU was significantly stronger among individuals with higher PIR levels compared to those with lower PIR, suggesting a potential socioeconomic influence on this relationship. Similarly, a more pronounced association between MHR and DFU was observed in White participants compared to non-White individuals, which may reflect ethnic variations in inflammatory or metabolic pathways. In contrast, the association between PHR and DFU remained consistent across all subgroups, indicating that this relationship is robust and independent of the demographic factors examined.

Since RBCs play a part in DFU wound repair, we investigated their potential mediation role in the relationship between DFU and HDL-related parameters. Blood is essential for wound healing, specifically, RBCs continuously provide oxygen, which supports cellular metabolism, encourages the synthesis of angiogenic growth factors and angiogenesis in diabetic wounds, and reduces the expression of proinflammatory cytokines [33, 34]. RBCs were validated as a mediating variable in the association between NHR and DFU by our mediation analysis. A substantial average causal mediation effect was found, suggesting that RBCs have some protective impact against DFU. The entire effect was 18.47% attributable to this mediated route. Crucially, a noteworthy average direct effect was also noted, suggesting that some of the relationship between NHR and DFU occurs via mechanisms other than RBCs.

Our study has several strengths. First of all, it is the first study to investigate the role of RBCs as a mediator and the association between HDL-related inflammatory markers and the incidence of DFU. Second, this study used nationally representative NHANES data, which ensures the results’ external validity and generalizability by covering several years of follow-up. Thirdly, the use of HDL-related indices as an easily accessible and reasonably priced laboratory test adds to the abundance of data available for in-depth research. However, some limitations do exist in this study. The existence of unmeasured variables can nevertheless have an impact on the analytical results even after careful confounding factor modifications. Additionally, the study could not establish a causal association between HDL-related inflammatory indicators and DFU prevalence.

### Clinical implications and future directions

The findings from this study carry several potential implications for the clinical management of DFU. The HDL-related inflammatory indices, NHR, MHR, and PHR, may serve as accessible and cost-effective biomarkers for improving risk stratification and early identification of DFU among patients with diabetes. Specifically, these indices could be integrated into routine metabolic panels to enhance screening protocols, allowing clinicians to identify high-risk individuals who may benefit from more intensive foot surveillance, preventive education, or targeted anti-inflammatory interventions.

Moreover, the mediating role of RBCs in the association between NHR and DFU suggests potential avenues for novel therapeutic strategies. Improving erythrocyte function and oxygen delivery, for instance, through nutritional supplementation or medications that enhance microcirculation, may help mitigate ulcer risk in susceptible patients.

Future research should prioritize longitudinal and interventional studies to establish causal relationships between these biomarkers and DFU outcomes. Additionally, exploring the underlying mechanisms could unveil new therapeutic targets. In this context, innovative imaging techniques such as speckle tracking echocardiography could be valuable for non-invasively evaluating the degree of myocardial fibrosis in diabetic patients [35]. Implementing such methodologies in future studies may help elucidate the intricate mechanisms linking systemic inflammation, subclinical cardiac organ damage, and the development of DFU, thereby providing a more comprehensive pathophysiological perspective. Validation in broader and more diverse populations is also essential to refine cutoff values and improve generalizability. Ultimately, these biomarkers may contribute to a more personalized and proactive approach to DFU prevention and management.

## Conclusions

Our findings show a clear link between neutrophils/high-density lipoprotein ratio, monocyte/high-density lipoprotein ratio, platelet/high-density lipoprotein ratio, and a higher prevalence of diabetic foot ulcer, with red blood cells mediating the interaction between neutrophils/high-density lipoprotein ratio and diabetic foot ulcer. These findings highlight the importance of monitoring HDL-related inflammatory indicators as biomarkers in the prevention and treatment of diabetic foot ulcer. Future prospective studies are required to determine the causal relationship and potential mechanisms linking HDL-related indices to diabetic foot ulcer development.

## Supplementary Information

Supplementary Material 1

## Data Availability

All the data are available upon reasonable request.
