# Supplementary material for: Association of high-density lipoprotein-related inflammatory indicators with diabetic foot ulcer in patients with diabetes: a population-based study
Source: Diabetol Metab Syndr. 2025 Sep 23;17:369. doi: 10.1186/s13098-025-01962-8 (PMC12455812; doi:10.1186/s13098-025-01962-8)
Supplement: Supplementary file 1 — Supplementary Material 1 [file 13098_2025_1962_MOESM1_ESM.docx]

Table S1. Categorization of baseline variables.

| Variables | description |
| --- | --- |
| Race/Ethnicity |  |
| White | Non-Hispanic White |
| Non-White | Mexican American; Other Hispanic; Non-Hispanic Black; Other Race - Including Multi-Racial |
| Family PIR | A ratio of family income to poverty threshold |
| CVD |  |
| Yes | Having a history of congestive heart failure, heart attack, coronary heart disease, angina, or stroke |
| No | Not having a history of congestive heart failure, heart attack, coronary heart disease, angina, or stroke |
| Alcohol use | Question: In the past 12 months, how often did you drink any type of alcoholic beverage?  Answer: 0-365 days |

Table S2. Subgroup analysis on the association of NHR with diabetic foot ulcer.

| NHR | | | |
| --- | --- | --- | --- |
| Subgroup | OR(95%CI) | *P*value | *P* for interaction |
| Age |  |  | 0.052 |
| 40-64 | 1.27(1.11,1.46) | 0.0007 |  |
| 65-85 | 1.05(0.90,1.21) | 0.544 |  |
| Gender |  |  | 0.9331 |
| Male | 1.16(1.02,1.31) | 0.0187 |  |
| Female | 1.15(0.98,1.35) | 0.0882 |  |
| PIR |  |  | 0.0268 |
| 0.00-1.21 | 1.01(0.86,1.18) | 0.9056 |  |
| 1.22-2.62 | 1.28(1.07,1.52) | 0.0065 |  |
| 2.64-5.00 | 1.38(1.14,1.67) | 0.0011 |  |
| Race |  |  | 0.4196 |
| White | 1.23(1.06,1.42) | 0.0069 |  |
| Non-White | 1.13(1.00,1.28) | 0.0439 |  |
| Marital Status |  |  | 0.0456 |
| Married/Living with a partner | 1.27(1.12,1.44) | 0.0002 |  |
| Widowed/Divorced/Separated | 0.98(0.83,1.16) | 0.8019 |  |
| Never married | 1.15(0.76,1.73) | 0.5135 |  |

Table S3. Subgroup analysis on the association of MHR with diabetic foot ulcer.

| MHR | | | |
| --- | --- | --- | --- |
| Subgroup | OR(95%CI) | *P*value | *P* for interaction |
| Age |  |  | 0.2724 |
| 40-64 | 1.65(0.55,4.98) | 0.3713 |  |
| 65-85 | 4.00(1.25,12.79) | 0.0192 |  |
| Gender |  |  | 0.0463 |
| Male | 5.16(1.77,15.01) | 0.0026 |  |
| Female | 0.94(0.25,3.61) | 0.9289 |  |
| PIR |  |  | 0.6118 |
| 0.00-1.21 | 1.87(0.51,6.77) | 0.3432 |  |
| 1.22-2.62 | 4.50(0.90,22.60) | 0.0674 |  |
| 2.64-5.00 | 4.27(0.95,19.25) | 0.0586 |  |
| Race |  |  | 0.1172 |
| White | 6.28(1.78,22.18) | 0.0043 |  |
| Non-White | 1.72(0.61,4.86) | 0.3054 |  |
| Marital Status |  |  | 0.0537 |
| Married/Living with a partner | 5.38(1.83,15.82) | 0.0022 |  |
| Widowed/Divorced/Separated | 0.87(0.24,3.13) | 0.8306 |  |
| Never married | 32.57(0.20,5387.18) | 0.1814 |  |

Table S4. Subgroup analysis on the association of PHR with diabetic foot ulcer.

| PHR | | | | |
| --- | --- | --- | --- | --- |
| Subgroup | OR(95%CI) | | *P*value | *P* for interaction |
| Age |  | |  | 0.2415 |
| 40-64 | 1.00(1.00,1.01) | | 0.0158 |  |
| 65-85 | 1.00(1.00,1.00) | | 0.5587 |  |
| Gender |  | |  | 0.803 |
| Male | 1.00(1.00,1.01) | | 0.0587 |  |
| Female | 1.00(1.00,1.01) | | 0.2317 |  |
| PIR |  | |  | 0.1747 |
| 0.00-1.21 | 1.00(1.00,1.00) | | 0.8216 |  |
| 1.22-2.62 | 1.00(1.00,1.01) | | 0.0412 |  |
| 2.64-5.00 | 1.01(1.00,1.01) | | 0.0185 |  |
| Race |  | |  | 0.788 |
| White | 1.00(1.00,1.01) | | 0.1648 |  |
| Non-White | 1.00(1.00,1.01) | | 0.0572 |  |
| Marital Status | |  |  | 0.2092 |
| Married/Living with a partner | 1.00(1.00,1.01) | | 0.0067 |  |
| Widowed/Divorced/Separated | 1.00(1.00,1.00) | | 0.9166 |  |
| Never married | 1.00(0.99,1.01) | | 0.5785 |  |
